# The potential habitat of *Phlomoides rotata* in Tibet was based on an optimized MaxEnt model

**DOI:** 10.3389/fpls.2025.1560603

**Published:** 2025-06-03

**Authors:** Jun-Wei Wang, Min Xu, Norbu Ngawang, Yonghao Chen, Ngawang Bonjor, Xiaoyan Jia, Zhefei Zeng, La Qiong

**Affiliations:** ^1^ Key Laboratory of Biodiversity and Environment on the Qinghai-Tibetan Plateau, Ministry of Education, School of Ecology and Environment, Tibet University, Lhasa, Xizang, China; ^2^ Yani Observation and Research Station for Wetland Ecosystem, Tibet University, Nyingchi, Xizang, China; ^3^ Forestry Survey and Planning Research Institute of Tibet Autonomous Region, Lhasa, Xizang, China

**Keywords:** *Phlomoides rotata*, MAXENT model, climate change, suitable habitat prediction, Tibetan medicinal plants

## Abstract

**Introduction:**

*Phlomoides rotata*, an important Tibetan medicinal plant, has garnered significant attention due to its remarkable medicinal value and ecological functions. However, overharvesting and climate change have progressively reduced its distribution range, threatening its survival.

**Methods:**

This study employed an optimized MaxEnt model, integrating field survey data and multiple environmental variables, to predict and analyze the potential suitable distribution of *P. rotata* in Tibet.

**Results:**

The model achieved high predictive accuracy, with Ture skill statistic (TSS) = 0.87 and Cohen’s Kappa Coefficient (Kappa) = 0.81. Under current climatic conditions, the suitable habitat area of *P. rotata* is 33.31×10^4^ km², primarily distributed in alpine meadows and sparse shrublands in regions such as Lhasa, Nyingchi, Qamdo, Shannan, and eastern Nagqu. Analysis of key environmental factors revealed that land cover type (30.7%), temperature seasonality (19.9%), and vegetation type (10.2%) are the most significant drivers influencing the distribution of *P. rotata*. Under future climate change scenarios, the distribution of suitable habitats exhibits notable dynamic trends. In the low-emission scenario (SSP126), the suitable habitat area shows an overall expansion. In contrast, under medium- and high-emission scenarios (SSP245 and SSP585), the suitable habitat area gradually shrinks. The distribution centers consistently migrate northwestward, with the longest migration distance observed under SSP585 (89.55 km).

**Discussion:**

This study identifies the critical driving factors for the distribution of *P. rotata* and elucidates its response patterns to climate change. These findings provide a theoretical foundation for the resource management, ecological conservation, and sustainable utilization of Tibetan medicinal plants while offering valuable references for the study of other alpine plants.

## 1 Introduction

Tibetan medicinal plants form a fundamental pillar of the traditional Tibetan medical system, valued for their diverse medicinal properties and distinctive therapeutic efficacy (Renzhen et al., 2022). Among these, *P. rotata*, a perennial herb endemic to the Qinghai-Tibet Plateau and a member of the genus Phlomoides in the Lamiaceae family, is a significant traditional Tibetan medicine known as “Dabuba” in Tibetan, with Tibet as its main production area (Liu et al., 2006). The plant is rich in medicinal chemical components and demonstrates significant pharmacological activities, such as enhancing blood circulation, reducing inflammation and pain, and mitigating rheumatoid arthritis (Li Z, et al., 2024; Wang et al., 2024; Zhou et al., 2023). In addition to its medicinal importance, *P. rotata* contributes significantly to alpine grassland ecosystems by preventing soil erosion and enhancing plateau environments, positioning it as an indispensable species for both ecological preservation and pharmaceutical applications (Zhao et al., 2022; Kala and Ratajc, 2012).

*P. rotata* thrives in unique habitats, primarily distributed in alpine grasslands and sparse shrub meadows at altitudes of 3,000–5,000 meters on the Qinghai-Tibet Plateau, where it faces multiple environmental stresses such as cold, intense ultraviolet radiation, and drought (Sun et al., 2012). However, *P. rotata* exhibits relatively low resistance to environmental stresses, and coupled with long-term overharvesting, its population size has gradually declined, resulting in its classification as a first-class endangered Tibetan medicinal plant and inclusion in the China Biodiversity Red List – Higher Plants Volume (Zhang et al., 2015). In recent years, with the intensification of climate change, significant alterations in climate and ecological conditions on the Qinghai-Tibet Plateau, such as rising temperatures and shifting precipitation patterns, have profoundly impacted the distribution, growth, and reproduction of regional plants. It is anticipated that many alpine species will adjust their geographic ranges in response to global warming (Shen et al., 2014). Research indicates that global warming could result in the reduction or shift of suitable habitats for alpine plants, representing a major threat to species population dynamics and the sustainable management of medicinal resources (Shen et al., 2014; Xu and Wu, 2024; Thuiller, 2007). Therefore, systematically assessing the suitable distribution range of *P. rotata* in Tibet and its response patterns to climate change is of great significance for conserving this rare medicinal resource, while also providing a scientific basis for optimizing its domestication and cultivation strategies.

Current research on *P. rotata* primarily focuses on its pharmacological activities, chemical constituents, and clinical applications (Li Z, et al., 2024; Wang et al., 2024; Zhou et al., 2023), while studies based on its geographic distribution and ecological characteristics remain relatively limited. Previous studies have attempted to use species distribution models (SDMs) to predict the potential distribution areas of *P. rotata* in China, the Qinghai-Tibet Plateau, and Sichuan Province, but these studies have several limitations: Firstly, the quality of distribution data is inconsistent, as it predominantly originates from specimen collections from the late 20th century, with limited GPS accuracy and reliability. Secondly, model parameters were not optimized and default settings were used directly, which could compromise the accuracy of the predictions. Thirdly, most studies relied solely on 19 bioclimatic variables, overlooking other ecological factors that might significantly influence the distribution of *P. rotata*, such as soil type, vegetation cover, and topography. Furthermore, limited correlation analysis among environmental variables might introduce biases into the model’s predictions. Lastly, regional variations in ecological adaptability were inadequately addressed, limiting the generalizability of the models within the intricate environmental conditions of the Qinghai-Tibet Plateau (Zhao et al., 2021; Xiong, 2022; Zhu et al., 2018; Wan et al., 2025).

Over recent years, the MaxEnt model (Maximum Entropy Model) has gained extensive use in predicting species’ ecological niches because of its superior predictive accuracy and resilience to incomplete data (Feng et al., 2019). By combining species distribution information with environmental variables, it identifies critical environmental drivers and forecasts potential suitable habitats under present and future climatic conditions (Elith et al., 2011; Phillips et al., 2006). Nonetheless, research employing the MaxEnt model to predict the distribution of *P. rotata* is scarce, mostly addressing large-scale analyses across China or the Qinghai-Tibet Plateau, while detailed studies specific to Tibet—the core area of its distribution—are absent, especially under climate change scenarios (Zhao et al., 2021; Wan et al., 2025). Therefore, this study systematically simulated and predicted the potential suitable distribution areas of *P. rotata* in Tibet using an optimized MaxEnt model, incorporating field survey data and diverse environmental variables. The primary objectives of this study are: (1) to identify the key environmental factors influencing the distribution of *P. rotata* in Tibet; (2) to predict its potential suitable distribution range and centroid migration patterns under current and future climate scenarios; and (3) to provide a scientific basis for the conservation, rational utilization, and sustainable development of *P. rotata* resources. The findings of this research assist in pinpointing optimal areas for the introduction and domestication of *P. rotata* while delivering theoretical foundations for the development and preservation of Tibetan medicinal flora. Additionally, they hold significant reference value in understanding the effects of global climate change on alpine plant species.

## 2 Materials and methods

### 2.1 Acquisition and processing of *P. rotata* sample data

**Figure 1:**
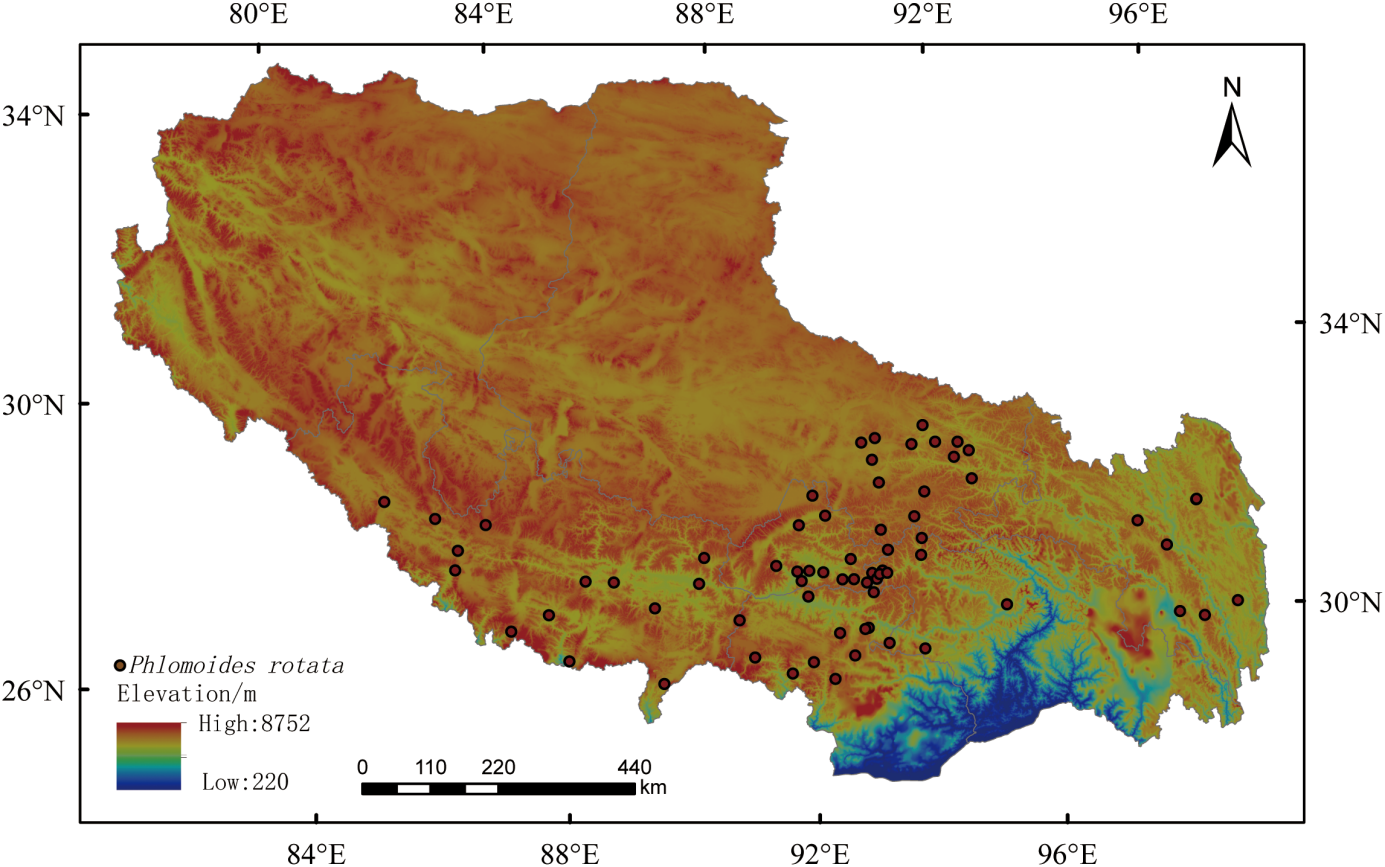
The distribution of sampling locations for *Phlomoides rotate*.

To investigate the ecological adaptability of *P. rotata*, a Tibetan medicine medicinal plant in the alpine regions of Tibet, this study adopts a species distribution modeling (SDMs) framework integrating field surveys and ecological factor analysis. To obtain detailed and accurate geographic distribution data of *P. rotata* within the Tibet Autonomous Region, our research team conducted field botanical surveys during the summer and autumn growing seasons from September 2020 to June 2024. A systematic survey was implemented across seven municipal districts in the Tibet Autonomous Region, yielding 69 georeferenced species occurrence points (**Figure 1**). To minimize sampling bias caused by clustering effects, we strictly controlled the straight-line distance between field sampling points, ensuring that the distance between any two points exceeded 10 km. This effectively reduced the impact of spatial autocorrelation on species distribution data. Finally, the 69 distribution data points for *P. rotata* were organized and converted into a.csv format file for use in constructing and simulating predictive analyses with the MaxEnt model.

### 2.2 Acquisition and processing of environmental factor data

The 19 bioclimatic variables used in this study were obtained from the WorldClim database (https://www.worldclim.org/), based on Shared Socioeconomic Pathways (SSP) provided by the sixth phase of the Coupled Model Intercomparison Project (CMIP6) using the BCC-CSM2-MR model. To explore the changes in suitable habitats for the Tibetan medicinal plant *P. rotata* under different climate scenarios in Tibet, we selected current and three future periods (2041–2060, 2061–2080, and 2080–2100), each under three climate scenarios (SSP126, SSP245, and SSP585). SSP126, SSP245, and SSP585 correspond to radiative forcing targets of 2.6 W/m², 4.5 W/m², and 8.5 W/m², respectively, representing different global development trajectories ranging from proactive climate policies to high-emission pathways (Wu et al., 2019; Fick and Hijmans, 2017; Zhang, 2018). Furthermore, data on topographic factors, including slope, aspect, and elevation, were sourced from the EarthEnv database (https://www.earthenv.org/) (Amatulli et al., 2018). Data on vegetation coverage percentages and land cover types were derived from the Global Maps database (https://globalmaps.github.io/). Soil factor data (D2, 20–40 cm soil layer) were obtained from the FAO’s Harmonized World Soil Database (https://www.fao.org/soils-portal/data-hub/soil-maps-and-databases/harmonized-world-soil-database-v20/en/). Data on land use were obtained from the National Tibetan Plateau Data Center (https://data.tpdc.ac.cn/home). Vegetation type (veg-class) and hydrological data were sourced from the China Center for Sustainable Development Database (https://www.resdc.cn/). All the above data were at a spatial resolution of 30 seconds (1 km × 1 km).

A total of 42 environmental variables were employed in this study, comprising 19 climatic factors, 16 soil factors, and 3 topographic factors, as well as vegetation coverage percentage, land cover type, land use data, vegetation type, and distance from to water systems (**Supplementary Table S1**). To minimize the influence of multicollinearity among environmental variables on the model’s predictive accuracy, the study referred to Zhao Guanghua’s methodology and conducted a multicollinearity analysis using the Variance Inflation Factor (VIF) via ArcGIS and SPSS software. The Variance Inflation Factor (VIF) is widely employed to assess multicollinearity among predictor variables (Zhao, 2020). Following established thresholds: VIF <10 indicates no substantial multicollinearity, 10≤VIF<100 suggests moderate multicollinearity, and VIF≥100 signifies severe multicollinearity issues (Zhao, 2020). Through integration of methodological recommendations from previous studies (Dormann et al., 2013) and preliminary experimental validation, variables demonstrating VIF values below 100 were retained as model inputs in this investigation.”

Through this screening process, 16 environmental factors were finalized for the ecological niche modeling and prediction of the potential suitable habitat of *P. rotata*. These included 5 bioclimatic factors, 4 soil factors, 3 topographic factors, as well as vegetation type, land cover type, vegetation coverage, and distance to water systems. To ensure the operability and comparability of the model, it was assumed that the 4 soil factors, 3 topographic factors, vegetation type, land cover type, vegetation coverage, and distance to water systems would remain unchanged across the three future time periods.

### 2.3 MaxEnt modeling and parameter optimization

The MaxEnt ecological niche modeling software used in this study was version 3.4.4 (http://biodiversityinformatics.amnh.org/open_source/MaxEnt/). ArcGIS version 10.5 (https://developers.ArcGIS.com/) and R software version 4.2.3 (https://www.r-project.org/) were also employed.

In this study, we adopted the method proposed by Cobos et al. and used the Kuenm package in R software to optimize two key parameters in MaxEnt settings: feature class (FC) and regularization multiplier (RM) (Cobos et al., 2019). According to the Akaike Information Criterion Coefficients (AICc), the lowest AIC value was achieved when the RM was set to 1.1 and the FC was LQP (**Supplementary Figure 1**). Therefore, this parameter combination was selected as the optimal model configuration for constructing the MaxEnt model to predict the potential distribution of *P. rotata*. In the MaxEnt model, 75% of the randomly selected distribution data was assigned to the training dataset, and the remaining 25% was designated as the testing dataset for model validation. Logistic output was selected for the model, and the jackknife method was employed to evaluate the contribution and permutation importance of each variable. The model was run 10 times in subsample mode, and the average of these repetitions was used as the final output (Liu et al., 2020; Santana et al., 2019; Oliaei et al., 2018).

### 2.4 Accuracy assessment of the MaxEnt model

To further evaluate the modeling results, this study calculated the TSS and Kappa values using the R package PresenceAbsence. The TSS evaluation criteria are as follows: TSS < 0.4, fail; 0.4 < TSS < 0.55, poor; 0.55 < TSS < 0.7, average; 0.7 < TSS < 0.85, good; TSS > 0.85, excellent (Monserud and Leemans, 1992; Wang, 2014; Swets, 1988). For Kappa, the evaluation standards are: Kappa < 0.4, poor; 0.4 < Kappa < 0.75, good; Kappa > 0.75, excellent (Zeiss et al., 2024).

### 2.5 Classification and centroids of suitable areas

The final predictive results obtained from running the MaxEnt model were imported into ArcGIS 10.5 software, where the reclassification tool was used to manually classify the potential suitable areas for *P. rotata*. The corresponding spatial area was calculated, and the results were visually displayed. The Maximum Test Sensitivity Plus Specificity Threshold (MTSPS) is a standard criterion for defining the boundaries of species’ suitable habitats. According to this method, areas with prediction probabilities ranging from 0 to the MTSPS value were classified as unsuitable, while areas with probabilities from the MTSPS value to 1.0 were classified as suitable (Wen et al., 2024; Wang et al., 2024). In this study, the MTSPS value was determined to be 0.2027. Therefore, areas with prediction probabilities (P) ≤ 0.2027 were classified as unsuitable for *P. rotata*, whereas areas with P > 0.2027 were classified as suitable. Based on this classification standard, the study further analyzed the distribution and spatial characteristics of the suitable habitat for *P. rotata*.

Additionally, the “Centroid Changes (Lines)” analysis method in the SDM Toolbox (v2.4) of ArcGIS 10.5 was used to calculate the centroid position and spatial change trends of the suitable habitat for *P. rotata*. Meanwhile, the “Distribution Changes Between Binary SDMs” analysis method in the SDM Toolbox was used to compare the area and range changes of the suitable habitat under current and future climate scenarios, obtaining dynamic changes in the expansion and contraction of *P. rotata*’s suitable habitat in Tibet during future periods.

## 3 Results and analysis

### 3.1 Model accuracy evaluation

Based on the optimized model parameter settings, the MaxEnt model prediction results showed a TSS value as high as 0.87 and a Kappa value of 0.81. According to the evaluation criteria, both values are classified as excellent.

### 3.2 Primary environment variables

**Figure 2:**
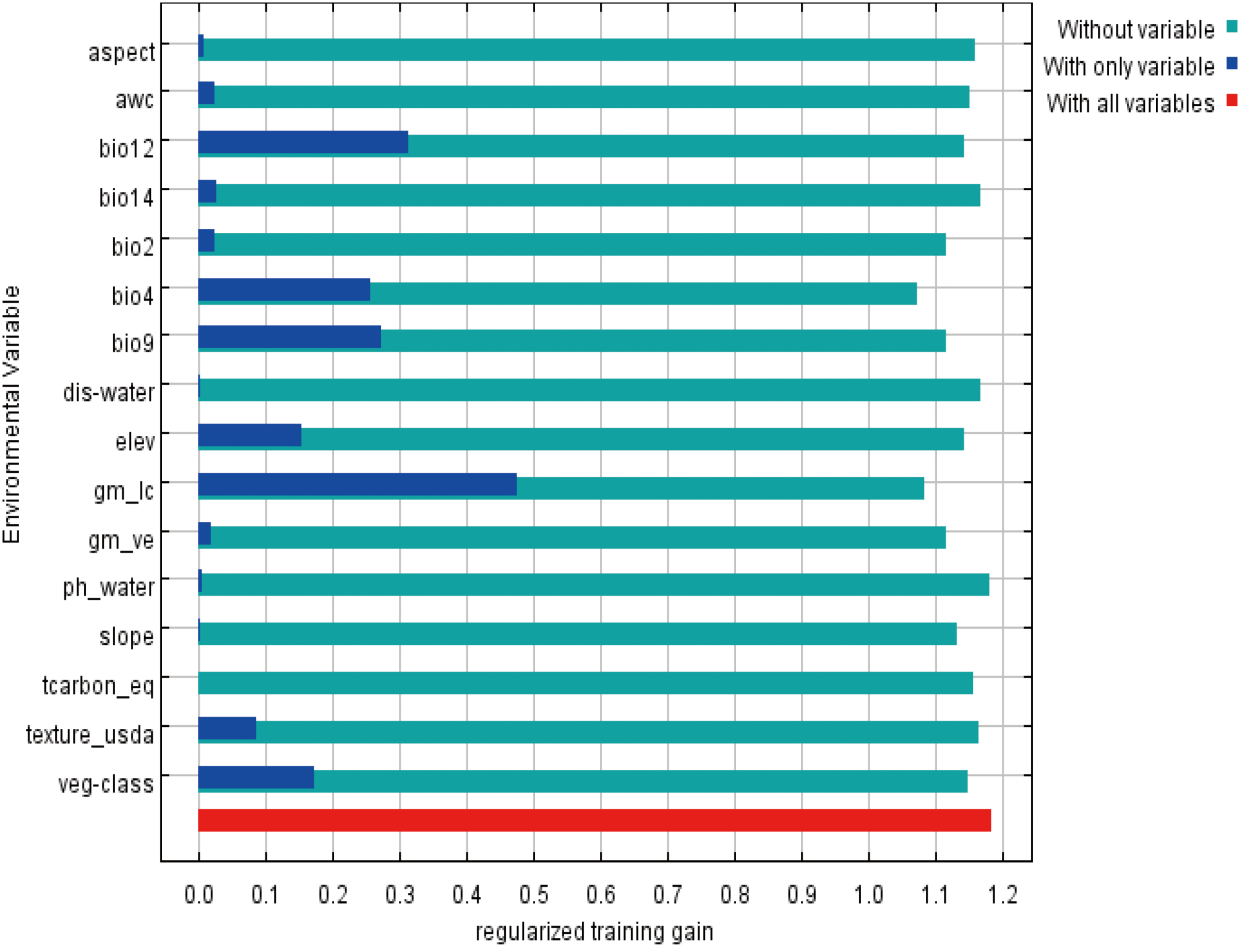
Jackknife test results for the importance of environmental variables in the MaxEnt model.

Among the 17 environmental variables ultimately used for model prediction, those with a contribution rate greater than 5.5% included land cover type (30.7%), bio4 (temperature seasonality, 19.9%), vegetation type (10.2%), bio2 (mean diurnal range/°C, 6.1%), soil texture classification (5.9%), and vegetation coverage (5.7%) (**Table 1**). These six environmental variables accounted for nearly 80% of the total contribution to the model prediction. Among them, land cover type (30.7%), temperature seasonality (19.9%), and vegetation type (10.2%) were the dominant variables influencing the distribution of *P. rotata*, with a combined contribution exceeding 60%. Additionally, the jackknife test of variable importance indicated that land cover type, bio4 (temperature seasonality), vegetation type, bio2 (mean diurnal range/°C), soil texture classification, and vegetation coverage were well-fitted to the training data. This demonstrates that these variables contained the most useful information not captured by others, further confirming that they are critical environmental factors influencing the geographic distribution of *P. rotata* in Tibet, playing a decisive role (**Figure 2**).

**Table 1:** Contributions and significant values of environmental factors.

Variable	Percent contribution (%)	Permutation importance (%)
Land cover type	30.7	8
Bio4	19.9	24.9
Vegetation type	10.2	3.3
Bio2	6.1	10.2
Soil texture classification	5.9	0.9
Vegetation coverage	5.7	7.7

**Figure 3:**
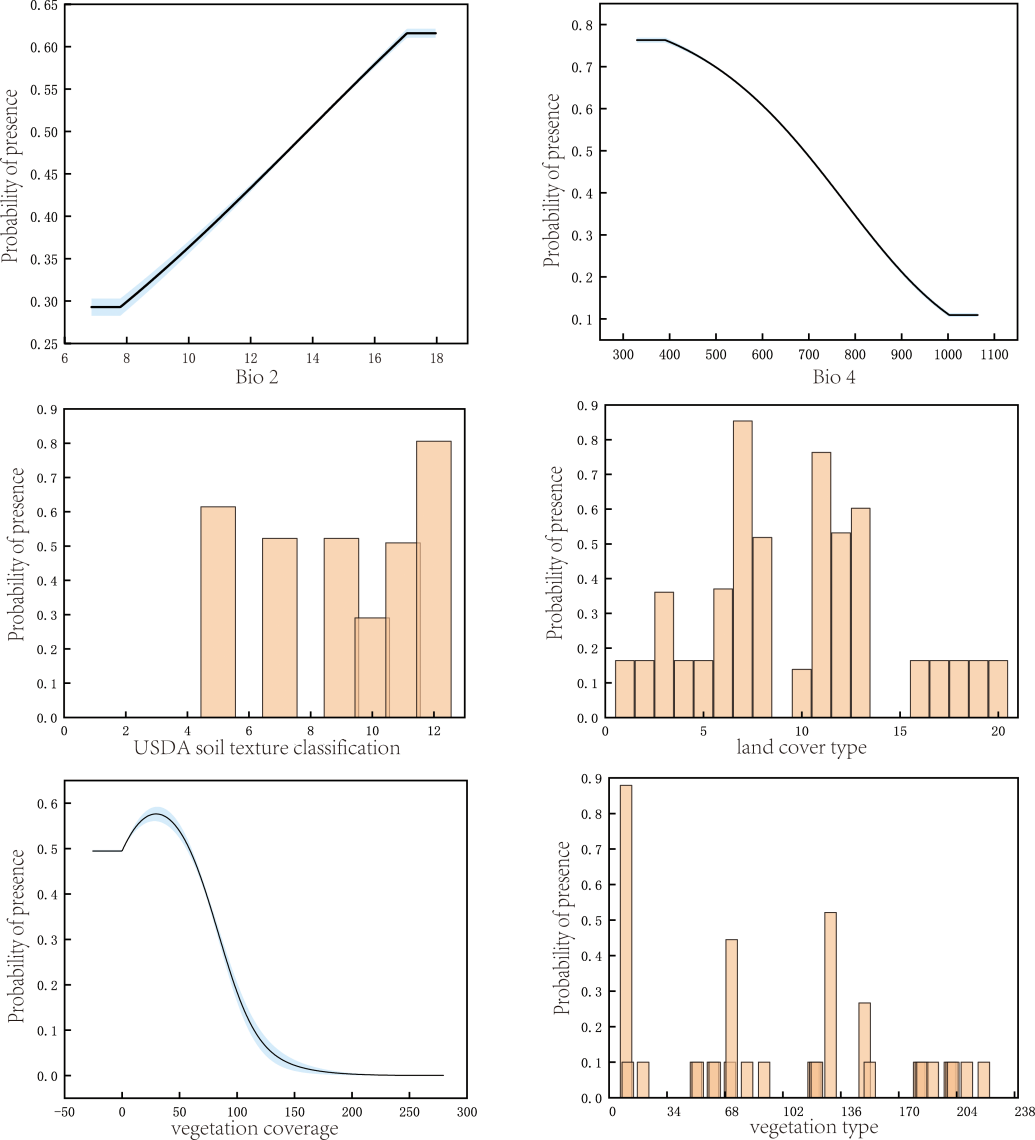
Response of *P. rotata* occurrence probability to key environmental variables.

A probability of occurrence greater than or equal to 0.5 was considered indicative of optimal environmental conditions for the survival of *P. rotata*. From the single-factor response plots (**Figure 3**), it can be observed that *P. rotata* thrives under the following environmental conditions: land cover type is optimal as herbaceous and shrubland; bio4 (temperature seasonality) is ≤ 689.38; vegetation type is classified as temperate deciduous broadleaf or coniferous forest; bio2 (mean diurnal range/°C) is ≥ 13.83°C; soil texture is categorized as clay loam, silt loam, loam, or loamy sand; and vegetation coverage ranges from 1.12% to 58.12%. These results are consistent with the ecological growth habits of *P. rotata*. Moreover, field investigations reveal that *P. rotata* is frequently found in moist alpine meadows, alpine shrub meadows, and under subalpine deciduous broadleaf forests with moderate canopy gaps, corroborating the precision and dependability of the model’s predictions.

### 3.3 Current and future suitable habitats of *P. rotata* in Tibet under different climate scenarios

Under current climatic conditions, *P. rotata* exhibits a suitable habitat area of 33.31×10^4^ km², primarily distributed across central, south-central, southwestern, eastern, and northeastern Tibet, including Lhasa, Shannan, Xigaze, Qamdo, and southeastern Nagqu regions. Projected changes in suitable habitat areas under future climate scenarios are as follows:

Under the low-emission SSP126 scenario, the suitable area expands from 33.31×10^4^ km² to 37.64×10^4^ km² during 2041–2060, subsequently contracts to 35.95×10^4^ km² by 2061–2080, and ultimately re-expands to 38.96×10^4^ km² by 2081-2100. This scenario demonstrates a persistent surplus over current habitat area, following a “expansion-minor contraction-re-expansion” dynamic pattern.

In contrast, the medium-emission SSP245 scenario reveals an overall contraction trend: the suitable area decreases from 33.31×10^4^ km² to 31.14×10^4^ km² by 2041-2060, temporarily recovers to 31.85×10^4^ km² during 2061-2080, and ultimately declines to 31.73×10^4^ km² by 2081–2100.

**Figure 4:**
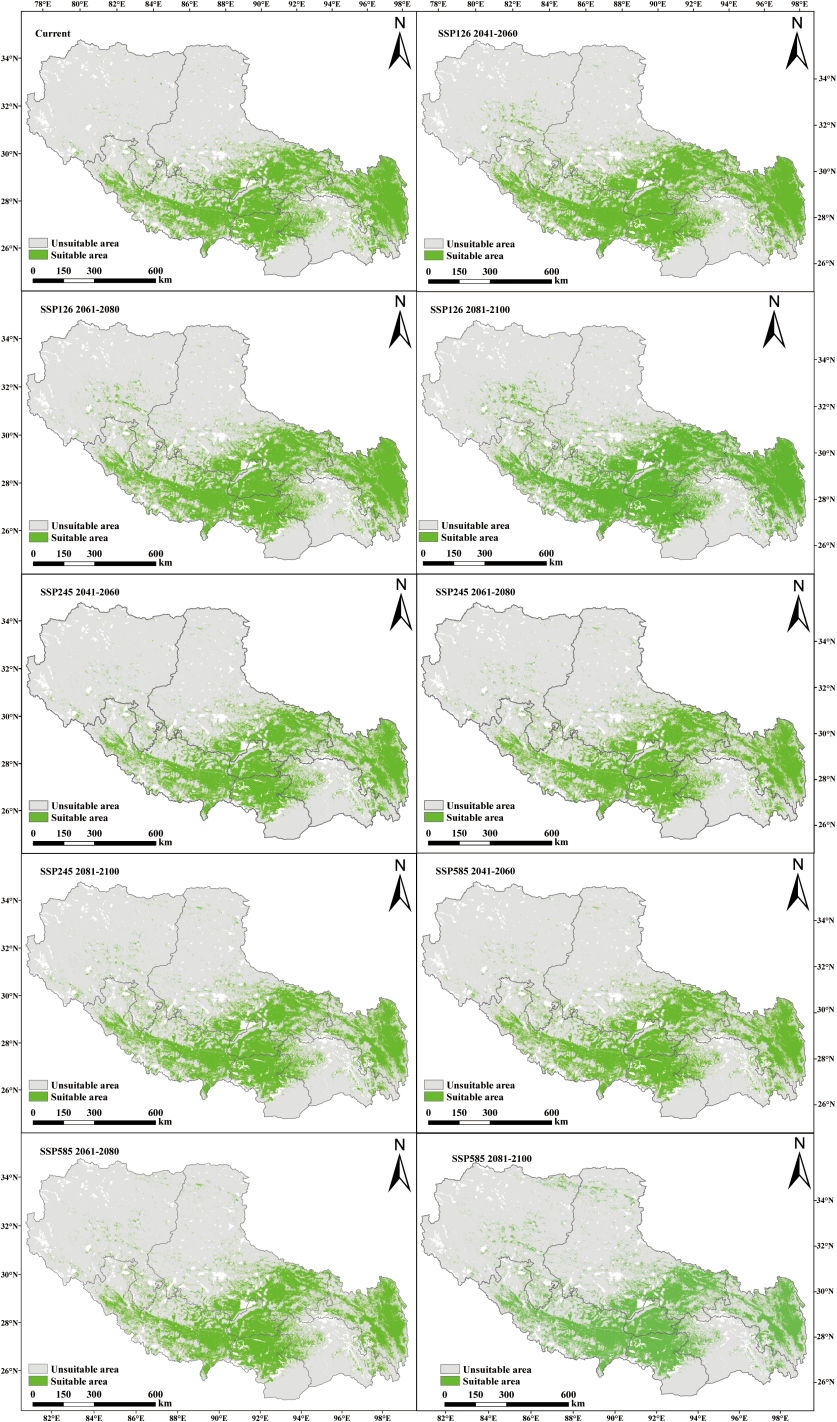
Prediction of potential suitable areas of *P. rotata* in Tibet in different periods.

The high-emission SSP585 scenario exhibits the most pronounced habitat loss, with the suitable area diminishing to 30.93×10^4^ km² by 2081–2100 (**Table 2**). Comparative analysis indicates that only SSP126 projections consistently exceed current habitat areas, while both SSP245 and SSP585 scenarios show net losses (**Figure 4**; **Table 2**). Spatially, central and eastern Tibet remain core suitable regions across all scenarios, with potential emergence of new habitat patches in northwestern high-altitude zones (e.g., northern Ali and Nagqu), which is closely associated with latitudinal gradient changes in regional climate regimes.

**Table 2:** The area of suitable areas of *P. rotata* in different periods.

Period	Suitable areas		Unsuitable areas	
	Proportion(%)	Area(10^4^km2)	Proportion(%)	Area(10^4^km^2^)
Current	27.70	33.31	72.30	86.97
2041-2060-SSP126	31.29	37.64	68.71	82.64
2041-2060-SSP245	25.89	31.14	74.11	89.14
2041-2060-SSP585	25.64	30.84	74.36	89.44
2061-2080-SSP126	29.89	35.95	70.11	84.33
2061-2080-SSP245	26.48	31.85	73.52	88.43
2061-2080-SSP585	25.25	30.38	74.75	89.90
2081-2100-SSP126	32.39	38.96	67.61	81.32
2081-2100-SSP245	26.38	31.73	73.62	88.55
2081-2100-SSP585	25.71	30.93	74.29	89.35

### 3.4 Future dynamic changes in the suitable habitat area of *P. rotata*

The results demonstrate distinct dynamic changes in the suitable habitat area of *P. rotata* under different climate scenarios:

Under the low-emission SSP126 scenario, the suitable habitat area expanded by 4.32×10^4^ km² during 2041–2060, contracted by 1.69×10^4^km² by 2061–2080, and subsequently re-expanded by 3.01×10^4^ km² by 2081–2100. This pathway consistently maintained a total habitat area exceeding current baseline levels, indicating potential beneficial effects of gradual climatic changes on species distribution.

The medium-emission SSP245 scenario exhibited a differentiated evolutionary pattern: the habitat area decreased by 2.18×10^4^ km² during 2041–2060, slightly increased by 0.71×10^4^ km² by 2061–2080, and marginally contracted again by 0.12×10^4^ km² by 2081–2100. Overall, this scenario showed a mild declining trend in habitat suitability.

The high-emission SSP585 scenario displayed the most pronounced negative response: the habitat area decreased by 2.47×10^4^ km² during 2041–2060 and 0.46×10^4^ km² by 2061–2080, followed by a partial recovery of 0.55×10^4^ km² by 2081–2100. Compared to current conditions, this scenario resulted in a net habitat contraction.

**Figure 5:**
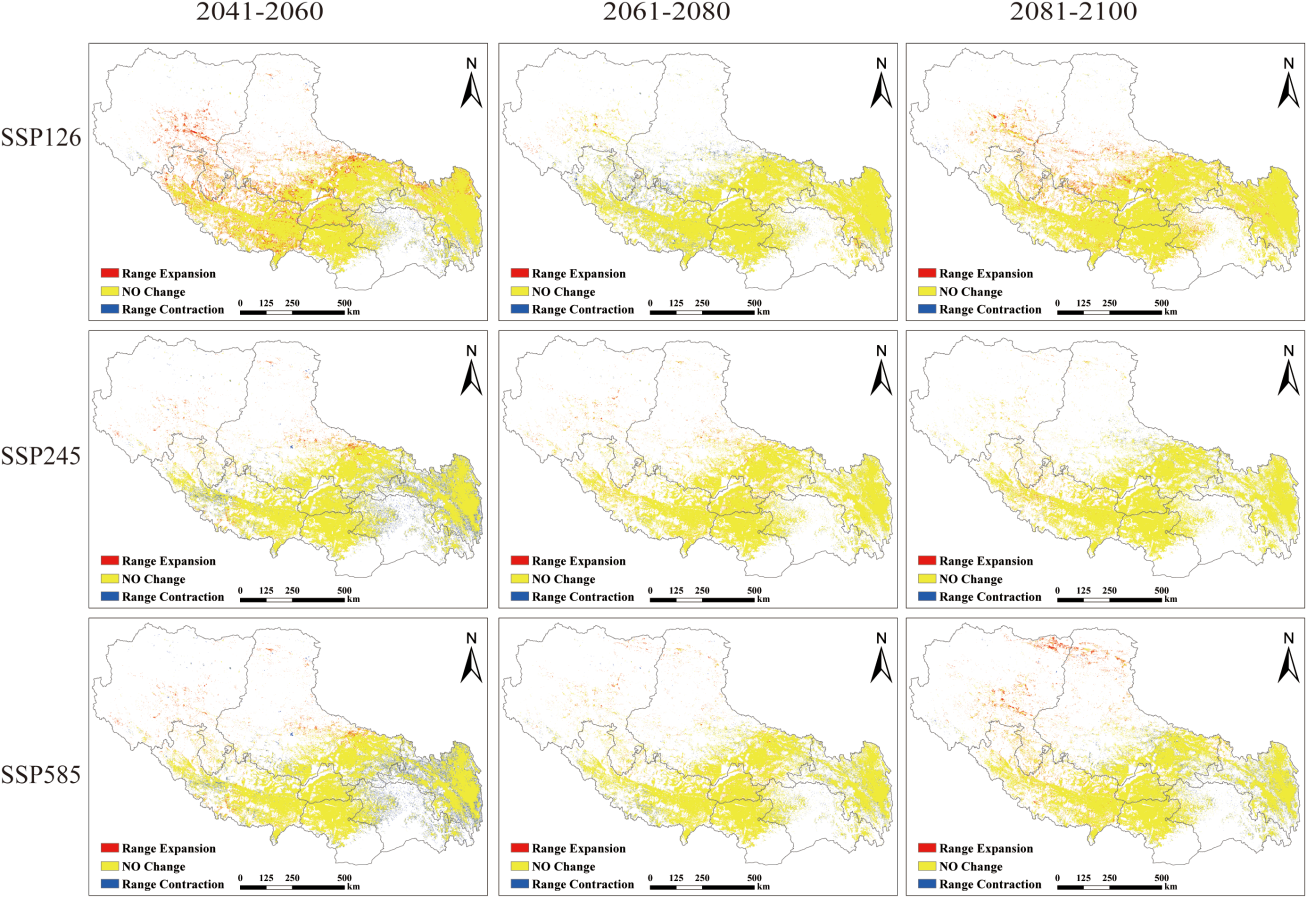
The spatial distribution change of suitable area of *P. rotata* in different periods.

Comparative analysis revealed habitat expansion exclusively under the SSP126 scenario, while both SSP245 and SSP585 scenarios projected net habitat losses (**Table 3**). Spatially, habitat gains predominantly occurred in the Nagqu and Ali regions, whereas contractions were concentrated in Nyingchi and Qamdo regions. Under all climate change scenarios (SSP126, SSP245, SSP585), cumulative habitat losses progressively intensified across the three time periods, with the majority of Tibet’s suitable areas exhibiting contraction trends over time (**Figure 5**). These findings suggest that L. rotata may face more substantial habitat contraction under future climate change, particularly under high-emission scenarios, necessitating prioritized monitoring of ecological responses and distributional shifts in these contexts.

**Table 3:** The suitable distribution area changes of *P. rotata* in different periods.

Scenarios	Period	Aggregate change	Expansions	Unchanged	Contractions
SSP126	2041-2060	4.32	5.26	32.37	0.94
	2061-2080	-1.69	0.38	35.56	2.07
	2081-2100	3.01	3.16	35.80	0.15
SSP245	2041-2060	-2.18	0.93	30.21	3.11
	2061-2080	0.71	1.18	30.67	0.47
	2081-2100	-0.12	0.63	31.10	0.75
SSP585	2041-2060	-2.47	1.07	29.77	3.54
	2061-2080	-0.46	0.84	29.54	1.30
	2081-2100	0.55	1.94	28.99	1.39

**Figure 6:**
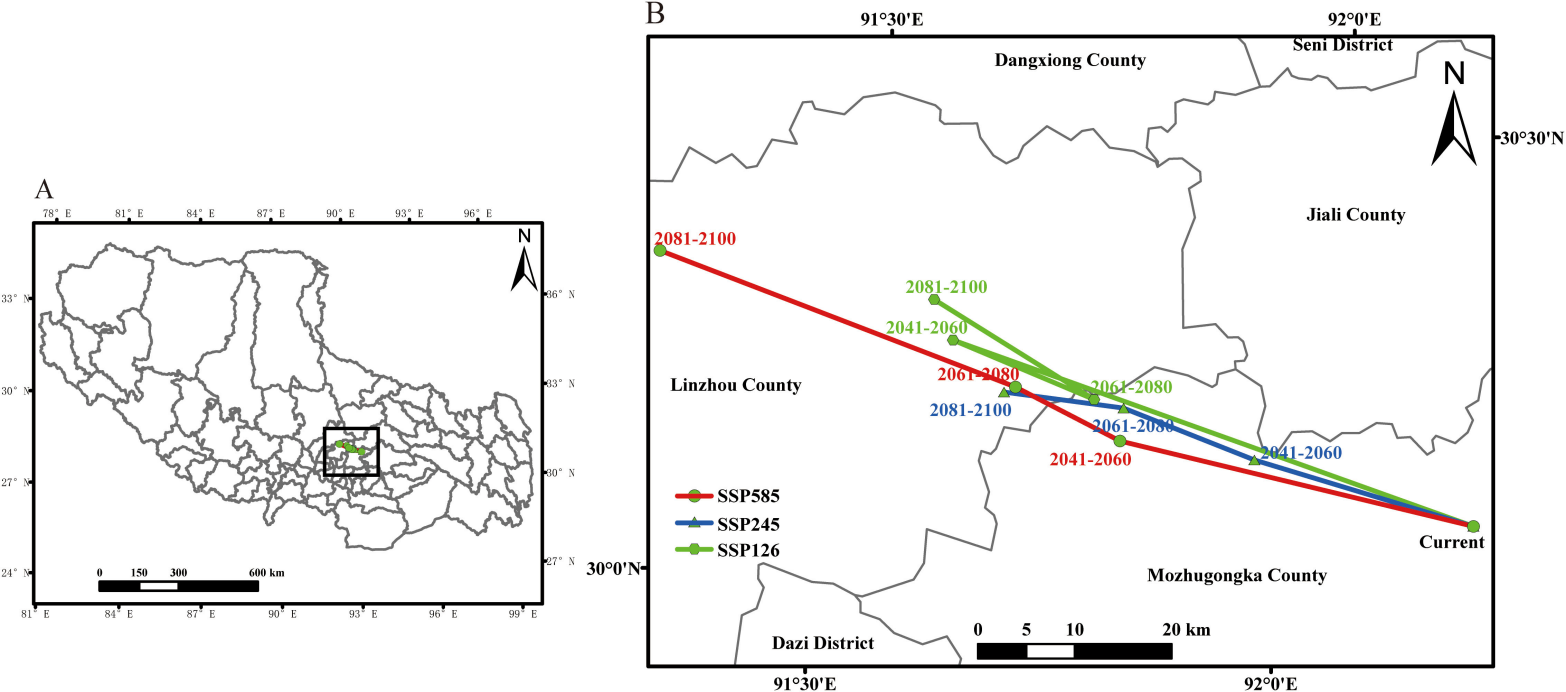
The geographical distribution change of the centroid of Lamiophlomis rotata suitable area under different climate scenarios **[(B)** is the expansion of part **(A)]**.

From the perspective of spatial shift direction, the changes in suitable habitats of *P. rotata* differ across climate scenarios, but the overall migration trend remains consistent, showing a general southeast-to-northwest migration pattern (**Figure 6**). In the current period, the center of the suitable habitat for *P. rotata* in Tibet is located in Mozhugongka County, Lhasa (92.197°E, 30.141°N). Under the SSP126 scenario, by 2081–2100, the center of the suitable habitat for *P. rotata* shifts northwestward to Linzhou County, Lhasa (91.585°E, 30.283°N), covering a migration distance of 60.89 km. However, during the mid-term period (2041–2060 to 2061–2080), the center of the suitable habitat experiences a brief reverse migration, shifting from northwest to southeast, before resuming a northwestward migration from 2061–2080 to 2081–2100.

Under the high-emission SSP585 scenario, the centroid of *P. rotata*’s suitable habitat is projected to shift northwestward by 2081–2100, relocating to Llinzhou County, Lhasa City (91.282°E, 30.294°N), an area proximate to the boundary of Dangxiong County, Lhasa, with a total displacement of 89.55 km. A comprehensive comparison of the migration distances under different climate scenarios indicates that by 2081–2100, the migration distances follow the order SSP585 > SSP126 > SSP245. This suggests that under the highest carbon emission scenario (SSP585), climate change has a more pronounced impact on the northwestward migration of *P. rotata*’s suitable habitat in Tibet.

## 4 Discussion

In this study, we used optimized MaxEnt model parameters to predict the potential suitable habitat area of *P. rotata* and evaluated the model’s accuracy by calculating TSS and Kappa values using the R package PresenceAbsence. The results showed a TSS value of 0.87 and a Kappa value of 0.81, indicating excellent predictive performance and high reliability of the model predictions. Field surveys further validated the model’s accuracy, revealing that *P. rotata* is mainly distributed in alpine shrublands and meadows, while areas with high vegetation coverage are unsuitable for its growth. Related studies have also demonstrated that the germination rate of *P. rotata* seeds is low in dark conditions, with light being a critical factor for its growth. High vegetation coverage can block light, thereby restricting its growth (Peng et al., 2018). High vegetation coverage blocks light, aligning with our findings. Additionally, different meadow types significantly influence the growth and reproduction of *P. rotata*, with the species thriving in alpine marshy meadows (Xie et al., 2021). Unlike previous studies where MaxEnt model predictions typically showed an expansion in medicinal plant distribution areas (Wen et al., 2024; Zhang et al., 2024), this study found that with intensified climate warming, the suitable habitat area for *P. rotata* may shrink to varying degrees in the future. This trend aligns with the sensitivity of alpine plants to climate change. Previous studies have shown that the distribution center of alpine meadow plant communities gradually shifts to higher altitudes with rising temperatures, leading to a reduction in suitable habitat area (Shen et al., 2014). The predictive results of this study further confirm this trend.

Environmental conditions are key drivers determining plant physiology, distribution, and phenology (Dong et al., 2023). This study found that under the context of climate change, the suitable habitat of *P. rotata* in Tibet exhibits a northwestward migration trend, with its primary distribution center located in the Lhasa region. The most influential environmental factors affecting its distribution include land cover type, temperature seasonality (bio4), vegetation type, mean diurnal range (bio2), soil texture classification, and vegetation coverage. Among these, land cover type (30.7%), temperature seasonality (19.9%), and vegetation type (10.2%) are the dominant factors, with a combined contribution exceeding 60%. Additionally, soil texture classification (5.9%) and vegetation coverage (5.7%) also play significant roles. The results suggest that the distribution of *P. rotata* is shaped by climatic factors (e.g., temperature and precipitation) as well as specific ecological traits, including vegetation type and land use, which reflect its sensitivity to microhabitat conditions. Further jackknife test results corroborated the importance of these factors in the model. Similarly, studies have indicated that the complex interactions between plant distributions and environmental variables significantly influence the suitable habitat areas of plants (Wen et al., 2024; Jia et al., 2024).

The prediction results indicate that the suitable distribution of *P. rotata* in Tibet is primarily located in alpine meadows and sparse shrub meadows in regions such as Lhasa, Nyingchi, Qamdo, Shannan, and eastern Nagqu. These areas exhibit relatively mild temperature seasonality and suitable soil and vegetation conditions, aligning closely with the ecological requirements of *P. rotata*. The dual importance of *P. rotata* in ecological conservation and medicinal value underscores the significance of studying its distribution changes. Firstly, under future climate change scenarios, it is crucial to protect its existing highly suitable habitats, particularly in regions such as Lhasa, Nyingchi, Qamdo, and Shannan, which should be prioritized for conservation. Secondly, potential expansion areas of suitable habitats in the future, such as northwestern Tibet and certain high-altitude regions, could serve as targets for scientific introduction and cultivation. This finding is consistent with studies on the future habitat expansion of high-value medicinal plants such as Lycium barbarum (Li J, et al., 2024), providing a reference for the cultivation planning of alpine plants.

Under future climate change scenarios (SSP126, SSP245, SSP585), the dynamic changes in the suitable habitat of *P. rotata* exhibit significant scenario dependence. Under the SSP126 scenario, the suitable habitat area generally shows an expansion trend, reaching 38.96×10^4^ km² by 2081–2100. However, under the high-emission scenarios SSP245 and SSP585, the suitable habitat area contracts, reducing to 31.73×10^4^ km² and 30.93×10^4^ km², respectively, by 2081–2100. Meanwhile, the distribution center of the suitable habitat consistently shows a significant northwestward migration trend, with the largest migration distance (89.55 km) occurring under the SSP585 scenario. This finding aligns with climate-driven response strategies observed in analogous alpine plant species (Li X, et al., 2024; Tang, 2024). As a characteristic alpine herb, *P. rotata* exhibits distribution patterns jointly constrained by summer heat stress and extreme winter cold. We posit that its northwestward range shift represents an adaptive response to global thermal changes, where rising temperatures exceed the species’ tolerance thresholds in its original habitats, forcing contraction toward cooler northwestern high-elevation and high-latitude refugia. Our projections demonstrate marked variations in suitable habitats under different emission scenarios (**Table 2**; **Figure 5**), highlighting climate-driven redistributions of this species. Under the low-emission scenario (SSP126), the suitable habitat of *P. rotata* shows an overall expansion trend, reaching its maximum extent by 2081–2100. Interestingly, during 2061–2080, the habitat exhibits a reverse migration. We hypothesize that the current suitable habitat of *P. rotata* has not fully occupied its ecological niche. Over time, new niches may emerge under the lowest carbon emission scenario, where its impact on suitable habitat is minimal. However, population density may later drive further northwestward habitat expansion. However, under medium and high-emission scenarios (SSP245 and SSP585), the suitable habitat shows an overall reduction trend, with a more pronounced contraction under the high-emission scenario. This indicates that the impact of climate warming on alpine plant habitats is significantly scenario-dependent, consistent with studies on other alpine plants, such as Primula elongata and its variants, which show that high-emission scenarios significantly compress their suitable distribution ranges (Bao et al., 2022; Yang et al., 2024).

Although future climate change may expand suitable areas in some regions, such as Nagqu and Ngari, the contraction of suitable habitats in Nyingchi and Qamdo may threaten the population stability of *P. rotata*. This indicates that alpine plants under climate change are at risk of habitat alteration and potential population declines caused by habitat fragmentation (Du et al., 2024). Future conservation efforts should prioritize the ecological response of *P. rotata* under high-emission scenarios and focus on maintaining the ecological integrity of its current suitable habitats. Additionally, the strong dependence of *P. rotata* on vegetation and soil characteristics indicates its high adaptability to microenvironmental conditions, but it also constrains the rapid expansion of its distribution.

While this study offers a systematic projection of *P. rotata*’s suitable habitats based on environmental variables such as climate and soil conditions, several limitations should be acknowledged. The predictions reflect theoretical distributions under climate suitability scenarios, whereas actual population distributions may be constrained by anthropogenic pressures. Specifically, the potential impacts of human activities (e.g., land-use changes, harvesting of Tibetan medicinal resources) and biotic factors (e.g., interspecific competition, disease dynamics) on habitat accessibility were not fully quantified. Furthermore, climate-driven shifts in ecological interactions (e.g., species co-occurrence networks, soil microbial community restructuring) remain unaddressed in the current modeling framework.

To comprehensively unravel the species’ response mechanisms to climate change, future research should integrate spatially explicit anthropogenic datasets (e.g., human footprint indices) and ecological network analyses. Coupling dynamic vegetation models with species-specific physiological thresholds could further enhance the mechanistic representation of habitat suitability under evolving climate regimes.

## 5 Conclusion

This study investigated the habitat adaptation mechanisms and climate change responses of the Tibetan medicinal plant *Phlomoides rotata* in alpine ecosystems of Tibet by optimizing parameter configurations of the MaxEnt model. The key findings are summarized as follows:

Current Suitable Habitat: The species is predominantly distributed in central, south-central, southwestern, eastern, and northeastern Tibet, including regions such as Lhasa, Shannan, Xigaze, Qamdo, and southeastern Nagqu.

Future Habitat Trends: Under intensifying climate change scenarios, the suitable habitat area of *P. rotata* is projected to contract substantially, particularly under high-emission scenarios (e.g., SSP585).

Key Environmental Factors: Land cover type (30.7%), temperature seasonality (19.9%), and vegetation type (10.2%) emerged as the primary environmental determinants, accounting for a combined contribution exceeding 60% to the model’s predictive performance.

Range Shift Dynamics: A pronounced northwestward migration trend toward higher elevations was identified under SSP585, revealing an “altitude-latitude synergistic compensation” strategy adopted by alpine plants to mitigate warming impacts.

Thus, it was of great significance to use the Maxent model to predict the development and utilization of *P. rotata* germplasm resources in the face of climate change.

## Data Availability

The original contributions presented in the study are included in the article/**Supplementary Material**. Further inquiries can be directed to the corresponding author/s.
