# Supplementary material for: The potential habitat of Phlomoides rotata in Tibet was based on an optimized MaxEnt model
Source: Front Plant Sci. 2025 Jun 3;16:1560603. doi: 10.3389/fpls.2025.1560603 (PMC12170608; doi:10.3389/fpls.2025.1560603)
Supplement: Supplementary file 7 [file Table1.docx]

Supplementary Material

# Supplementary Table S1

Table S1 Preliminary list of variables

| Abbreviation | Variable |
| --- | --- |
| Bio1 | Annual mean temperature/℃ |
| Bio2 | Mean diurnal range/℃ |
| Bio3 | Isothermally [(Bio2/Bio7)×100] |
| Bio4 | Temperature seasonality |
| Bio5 | Maximum temperature of warmest month/℃ |
| Bio6 | Minimum temperature of coldest month/℃ |
| Bio7 | Temperature annual range (Bio5–Bio6)/℃ |
| Bio8 | Mean temperature of wettest quarter/℃ |
| Bio9 | Mean temperature of driest quarter/℃ |
| Bio10 | Mean temperature of warmest quarter/℃ |
| Bio11 | Mean temperature of coldest quarter/℃ |
| Bio12 | Annual precipitation/mm |
| Bio13 | Precipitation of wettest period/mm |
| Bio14 | Precipitation of driest period/mm |
| Bio15 | Precipitation seasonality (CV) |
| Bio16 | Precipitation of wettest quarter/mm |
| Bio17 | Precipitation of driest quarter/mm |
| Bio18 | Precipitation of warmest quarter/mm |
| Bio19 | Precipitation of coldest quarter/mm |
| Slope | Slope |
| Aspect | Aspect |
| elev | Elevation |
| Gm-ve | Vegetation coverage |
| Gm-lc | Land cover type |
| Veg-class | Vegetation type |
| Dis-water | Distance from water system |
| D*_AWC | Available water storage per unit of soil |
| D*_COARSE | Thickness of soil sand |
| D*_SAND | Soil sand content |
| D*_SILT | Soil silt content |
| D*_CLAY | Soil clay content |
| D*_TEXTURE_USDA | soil texture classification |
| D*_ORG_CARBON | organic carbon contents of soil |
| D*_PH_WATER | soil acidity and alkalinity |
| D*_TOTAL_N | soil total nitrogen content |
| D*_CN_RATIO | soil c/n ratio |
| D*_CEC_SOIL | Cation exchange capacity of soil |
| D*_CEC_CLAY | Cation exchange capacity of cohesive soils |
| D*_TEB | soil exchangeable base |
| D*_ESP | Soil exchangeable sodium salt |
| D*_TCARBON_EQ | Soil calcium carbonate content |
| D*_ELEC_COND | soil bulk electrical conductivity |
