# Supplementary material for: The potential habitat of Phlomoides rotata in Tibet was based on an optimized MaxEnt model
Source: Front Plant Sci. 2025 Jun 3;16:1560603. doi: 10.3389/fpls.2025.1560603 (PMC12170608; doi:10.3389/fpls.2025.1560603)
Supplement: Supplementary file 9 [file Table3.docx]

References.

<https://www.ecologica.cn/html/2020/19/stxb201908081666.htm>

The official documentation of the "presenceabsence" package.

[PresenceAbsence: Presence-Absence Model Evaluation](https://cran.r-project.org/web/packages/PresenceAbsence/PresenceAbsence.pdf)

**Reference Script.**

if (!requireNamespace("PresenceAbsence", quietly = TRUE)) {

install.packages("PresenceAbsence")

}

library(PresenceAbsence)

"One is the predicted values file (predictions.csv), and the other is the actual observation values file (observations.csv). These two files should contain the same number of rows and columns, and each row corresponds to a location."

# "Read the predictions file (which contains binary presence/absence predictions)."

predictions <- read.csv("predictions.csv", header = TRUE, row.names = 1)

# "Read the actual observations file (presence/absence)."

observations <- read.csv("observations.csv", header = TRUE, row.names = 1)

if (nrow(predictions) != nrow(observations) || ncol(predictions) != ncol(observations)) {

stop("The dimensions of the predicted values and the observation values are inconsistent. Please check the files.")

}

# "Convert the data into matrix form."

predictions_matrix <- as.matrix(predictions)

observations_matrix <- as.matrix(observations)

# "Calculate TSS and Kappa values."

evaluation_result <- pa.eval(predictions_matrix, observations_matrix)

tss_values <- evaluation_result$TSS

kappa_values <- evaluation_result$Kappa
